# Fixed-Dose Subcutaneous Methylnaltrexone in Patients with Advanced Illness and Opioid-Induced Constipation: Results of a Randomized, Placebo-Controlled Study and Open-Label Extension

**DOI:** 10.1089/jpm.2014.0362

**Published:** 2015-07-01

**Authors:** Janet Bull, Charles V. Wellman, Robert J. Israel, Andrew C. Barrett, Craig Paterson, William P. Forbes

**Affiliations:** ^1^ Four Seasons, Flat Rock, North Carolina.; ^2^ Hospice of the Western Reserve, Cleveland, Ohio.; ^3^ Progenics Pharmaceuticals, Inc., Tarrytown, New York.; ^4^ Salix Pharmaceuticals, Ltd., Raleigh, North Carolina.

## Abstract

***Background:*** Subcutaneous methylnaltrexone (MNTX), dosed based on body weight, is efficacious and well tolerated in inducing bowel movements in patients with advanced illness and opioid-induced constipation (OIC); however, fixed-dose administration of MNTX may improve ease of administration.

***Objective:*** The study objective was to assess safety and efficacy of fixed-dose MNTX in two phase 4 trials.

***Methods:*** In a double-blind, randomized, placebo-controlled trial (RCT), patients with advanced illness and OIC received MNTX (8 mg or 12 mg by body weight [38 kg to <62 kg or ≥62 kg, respectively]) or placebo every other day (QOD) for two weeks. Patients completing the RCT could enroll in an open-label extension (OLE) study with MNTX administered as needed (PRN). The primary endpoint was percentage of patients with a rescue-free bowel movement (RFBM) within four hours after ≥2 of the first 4 doses in the first week.

***Results:*** In the RCT, 116 and 114 patients received MNTX and placebo, respectively, and 149 patients continued to the OLE study. The percentage of patients achieving primary endpoint was 62.9% and 9.6% for MNTX and placebo groups, respectively (*p*<0.0001). Median time to RFBM after the first dose was 0.8 hour and 23.6 hours in MNTX and placebo groups, respectively (*p*<0.0001). Efficacy results during the OLE study were consistent with the RCT. MNTX demonstrated a favorable safety profile in the RCT and OLE study.

***Conclusion:*** Fixed-dose MNTX administered QOD in the RCT and PRN in the OLE study demonstrated robust efficacy and was well tolerated in treating OIC in patients with advanced illness.

## Introduction

Opioids are often prescribed to treat moderate to severe pain and dyspnea in patients with advanced illness, and constipation is a common and often distressing adverse effect of chronic opioid therapy.^1^ Opioid-induced constipation (OIC) is observed in up to 95% of patients with advanced illness taking long-term opioids.^2^ Severe OIC may result in opioid dose reduction or limit up-titration, leading to inadequate pain control.^3^ OIC can have a profound negative impact on patient quality of life and increase health care costs related to higher rates of hospital admissions, inpatient days, and emergency department visits.^4,5^

In most cases OIC is managed nonspecifically with stool softeners, osmotic agents, and stimulant laxatives.^6^ However, these approaches are often insufficient and burdensome, and there is no evidence of their efficacy for OIC in controlled clinical trials.^6–8^ In addition, these strategies do not target the underlying cause of OIC. Opioids cause OIC by inhibiting propulsive motor activity of the gastrointestinal tract, primarily via local μ-opioid receptors in the plexus myentericus, the complex neural network of the gut.^9–11^ Two strategies have been developed to specifically block local μ-opioid receptor activation by opioids: (1) enteral use of centrally acting μ-opioid receptor antagonists with limited systemic absorption and (2) administration of peripherally restricted μ-opioid receptor antagonists.^12–14^ Centrally acting μ-opioid receptor antagonists like naloxone and naltrexone can reverse OIC, but they may also negatively impact opioid analgesic effects because of their ability to cross the blood-brain barrier. However, peripherally restricted μ-opioid receptor antagonists have limited access to the central nervous system and therefore relieve OIC without compromising the centrally mediated effects of opioid analgesia.

Methylnaltrexone (MNTX), a peripherally restricted μ-opioid receptor antagonist and derivative of naltrexone, has restricted ability to cross the blood-brain barrier and antagonizes the undesirable opioid effects on the gut, such as delaying gastric emptying^15^ and prolonging oral-fecal transit time.^16^ The safety and efficacy of subcutaneous MNTX for inducing a bowel movement in patients with advanced illness and OIC and receiving palliative care have been evaluated in two double-blind, placebo-controlled, multicenter, phase 3 studies.^17,18^ These trials were performed using weight-based dosing of MNTX. Compared with weight-based dosing, fixed-dose administration of subcutaneous MNTX can simplify and improve ease of administration for patients and caregivers. Therefore, a randomized, placebo-controlled trial (RCT) and an open-label extension (OLE) study were conducted to determine the efficacy and safety of fixed-dose subcutaneous MNTX in patients with advanced illness and OIC in a variety of health care situations.

## Methods

In the double-blind, multicenter, two-week RCT (study 4000; ClinicalTrials.gov identifier NCT00672477), patients were randomly assigned (1:1) to receive subcutaneous MNTX (8 mg or 12 mg based on body weight 38 kg to <62 kg or ≥62 kg, respectively) or placebo administered every other day (QOD) in various clinical settings (e.g., inpatient, outpatient, acute care, long-term care, assisted living, home hospice, and skilled nursing facility). After a screening/baseline clinic visit, the treatment phase consisted of three scheduled visits: on day 1 (first dose), day 7 (dose three or four), and day 14 (or early termination). Patients treated with MNTX or placebo who completed the treatment phase of this study could enroll in an OLE study if they met eligibility criteria. Patients not enrolling in the OLE study had a follow-up visit 15 to 21 days after the last dose of the study drug. In the multicenter, 10-week OLE study (study 4001; ClinicalTrials.gov identifier NCT00672139), patients received MNTX based on body weight (8 mg or 12 mg for 38 kg to <62 kg or ≥62 kg, respectively) on an as-needed basis (PRN), but no more than one dose per day. In the OLE study, the baseline visit was followed by treatment phase visits at week 4 and week 10 (or early discontinuation). Patients also had a follow-up visit 15 to 21 days after the administration of last dose of MNTX in the OLE study. The protocol was approved by institutional review boards and ethics committees, and all patients provided written informed consent.

### Study population

Patients eligible for the RCT were ≥18 years of age with advanced illness (defined as a terminal illness [e.g., incurable cancer or other end-stage disease]), had a life expectancy of ≥1 month and OIC (<3 bowel movements in the last week and no bowel movement in 24 hours or no bowel movement in 48 hours), and were receiving stable doses of laxatives and opioids. Patients received opioids on a regular schedule for ≥2 weeks before the first dose of study drug, and a stable opioid regimen was defined as no reduction in opioid dose of ≥50% for ≥3 days prior to the first dose of study drug. Patients with any disease process suggestive of gastrointestinal obstruction or clinically significant active diverticular disease, fecal impaction, peritonitis, bowel surgery ≤10 days before dosing, or fecal ostomy, or with a body weight <38 kg were excluded. A stable laxative regimen was required for each patient ≥3 days before the first dose of the study drug and a baseline regimen was continued during the two-week treatment period as appropriate. Patients in the OLE study had similar eligibility requirements as in the RCT, had completed the RCT, and had an anticipated need for treatment of OIC during the 10-week study duration. The baseline laxative regimen administered during the RCT was continued during the 10-week OLE study as necessary. Patients were allowed to use rescue medications or undergo manual disimpaction procedure if needed, but not within four hours before or four hours after receiving a dose of the study drug in both the RCT and OLE study. Prohibited medications in the RCT and OLE study included tegaserod, lubiprostone, opioid antagonists or partial antagonists, and combination opioid and opioid antagonist products.

### Measures

#### Efficacy

Patients or their caregivers were provided diaries to record information daily on clinically notable bowel movements during the RCT and OLE study; the patients were to return their diaries to the study staff at each study visit. In the RCT, the primary efficacy endpoint was the percentage of patients with a rescue-free bowel movement (RFBM) within four hours after ≥2 of the first 4 doses (i.e., the first week of treatment). RFBM was defined as a bowel movement without use of any rescue medication or procedure within four hours before the bowel movement.

Secondary efficacy endpoints in the RCT included the percentage of patients with the first RFBM within four hours after the first dose, the percentage of patients with an RFBM within four hours after ≥4 of the maximum 7 doses, the number of bowel movements within 24 hours after dosing per week, the number of RFBMs within 24 hours after dosing per week, the percentage of patients using rescue laxatives, the time to first RFBM after the first dose of study drug, and the time to RFBM within 24 hours after each dose.

Efficacy endpoints in the OLE study were exploratory and included the number of bowel movements within 24 hours after dosing per week, the number of days with bowel movements within 24 hours of dosing per week, and the percentage of injections resulting in bowel movement within four hours of dosing.

#### Safety

Safety assessments included monitoring adverse events (AEs), clinical laboratory tests, vital signs, and concomitant medications throughout the treatment phase (days 1 to 14) and during the follow-up period (days 15 to 29) in the RCT and throughout the 10-week OLE study. In the RCT, patients rated current pain and worst pain in the preceding four hours (±30 minutes; on a scale of 0 [none] to 10 [worst possible pain]) at baseline, after first dose of study medication (day 1), and after the fourth dose of medication (generally on day 7).

### Statistical analyses

Demographics and baseline disease characteristics were summarized using descriptive statistics. Enrollment of 127 patients in each treatment group (*N*=254 patients) in the RCT was estimated to provide >90% power to detect a ≥20% difference between the MNTX and placebo groups for the primary efficacy endpoint (α=0.05; two-sided). In the RCT, primary and secondary efficacy endpoint analyses were conducted in the intent-to-treat population. Treatment groups were compared using a stratified Cochran-Mantel-Haenszel test, and baseline body weight (<62 kg versus ≥62 kg) was considered a major source of variability and was used as the stratification factor for all efficacy analyses in the RCT. The Fisher's exact test was used for primary efficacy endpoint comparisons of treatment groups based on baseline body weight (≤114 kg versus >114 kg). For the time-to-event endpoints (e.g., time to first RFBM), distribution of the event times was estimated using the Kaplan-Meier method. The safety population in both the RCT and the OLE study included all patients who received ≥1 dose of the study drug.

## Results

### Patient disposition and demographics

**FIG. 1.**
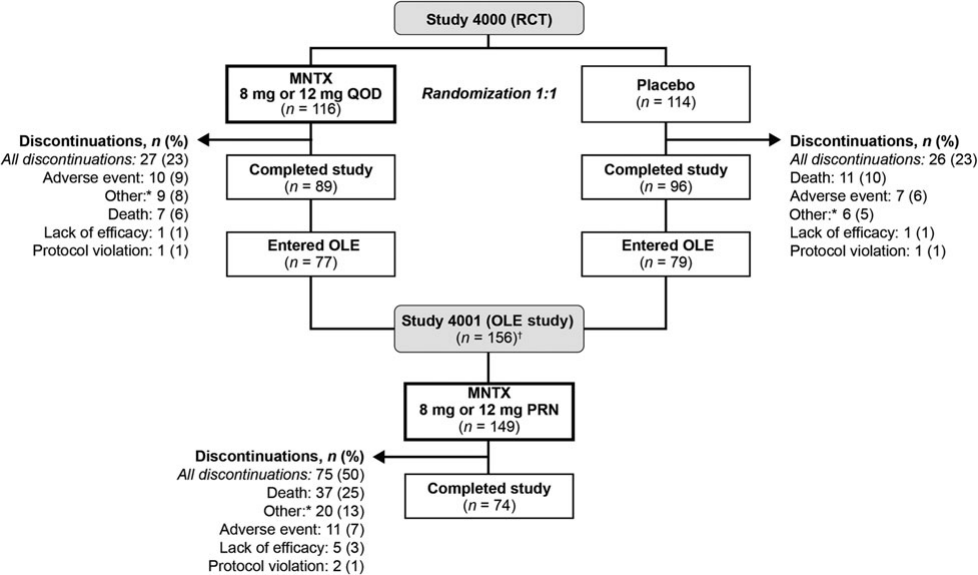
Patient disposition. MNTX, methylnaltrexone; OLE, open-label extension; RCT, randomized, placebo-controlled trial. *Other category includes discontinuation because of investigator and patient request. ^†^Seven patients were enrolled in the OLE study but did not receive any medication.

In the RCT, of the 237 patients randomized, 230 patients received ≥1 dose of the study drug (116 and 114 patients in the MNTX and placebo groups, respectively; see Fig. 1). Of the 156 patients entering the OLE study from the RCT, 149 received ≥1 dose of MNTX. Demographic and baseline characteristics were generally similar between treatment groups in the RCT (see Table 1). In the MNTX group, approximately two-thirds of the patients had a body weight of ≥62 kg and were treated with MNTX 12 mg/day. Five patients in each treatment group weighed >114 kg. The overall patient population (*n*=230) in the RCT was well balanced between males and females, and the patients were predominantly white (93.9%) with a mean age of 65.5 years. In addition, the most common underlying advanced illness was cancer (66.1%), the median daily morphine equivalent dose was 176.8 mg/d, and the mean duration of OIC was 76.6 weeks. There were no notable differences in demographic and baseline characteristics between the MNTX and placebo groups.

**Table 1.** Demographic and Baseline Characteristics for the RCT

*Characteristic*	*MNTX 8 mg or 12 mg QOD (*n*=116)*	*Placebo (*n*=114)*
Age, years, mean (SD)	65.3 (12.9)	65.7 (13.0)
Sex, *n* (%)		
Male	60 (51.7)	58 (50.9)
Female	56 (48.3)	56 (49.1)
Race, *n* (%)		
White	108 (93.1)	108 (94.7)
Black	5 (4.3)	3 (2.6)
Other	3 (2.6)	3 (2.7)
Primary diagnosis, *n* (%)		
Cancer	79 (68.1)	73 (64.0)
Pulmonary disease	14 (12.1)	13 (11.4)
Cardiovascular disease	13 (11.2)	11 (9.6)
Neurologic disease	4 (3.4)	3 (2.6)
Other	6 (5.2)	14 (12.3)
Duration of underlying advanced illness, years, mean (SD)	4.2 (6.0)	5.0 (7.0)
Morphine equivalent, mg/d		
Mean (SD)	369.5 (656.8)	404.6 (887.6)
Median (range)	180.0 (4.5–4427.0)	160.8 (9.0–7228.6)
Weight category, *n* (%)		
<62 kg	45 (38.8)	41 (36.0)
≥62 kg	71 (61.2)	73 (64.0)
Weight, kg		
Mean (SD)	72.2 (20.8)	73.4 (24.1)
Median (range)	68.0 (38.1–158.8)	68.5 (38.6–225.9)
Duration of OIC, weeks, mean (SD)	75.1 (152.9)	78.1 (227.4)
Number of BMs in the last seven days before first dose, mean (SD)	1.7 (0.9)	1.7 (0.9)
Prior laxative use, *n* (%)^a^		
Any laxative	116 (100.0)	114 (100.0)
Docusate sodium with senna	40 (34.5)	36 (31.6)
Bisacodyl	34 (29.3)	40 (35.1)
Lactulose	32 (27.6)	26 (22.8)
Polyethylene glycol 3350	28 (24.1)	30 (26.3)
Docusate sodium	21 (18.1)	25 (21.9)
Magnesium hydroxide	19 (16.4)	22 (19.3)
Sodium phosphate enema	13 (11.2)	10 (8.8)
Senna	12 (10.3)	22 (19.3)
Concomitant laxative use, *n* (%)^a^		
Any laxative	107 (92.2)	111 (97.4)
Docusate sodium with senna	38 (32.8)	37 (32.5)
Docusate sodium	18 (15.5)	28 (24.6)
Senna	16 (13.8)	27 (23.7)
Bisacodyl	14 (12.1)	24 (21.1)
Polyethylene glycol 3350	21 (18.1)	23 (20.2)
Lactulose	20 (17.2)	20 (17.5)

^a^ Patients could have taken ≥1 laxative.

BM, bowel movement; MNTX, methylnaltrexone; OIC, opioid-induced constipation; QOD, every other day; RCT, randomized, placebo-controlled trial; SD, standard deviation.

In the OLE study, similar to the RCT, the patient population was well balanced between males (48.3%) and females (51.7%), and patients were predominantly white (92.6%) with a mean age of 65.9 years. Ninety-two (61.7%) of the 149 patients were treated with MNTX 12 mg/day, and 57 (38.3%) patients were treated with MNTX 8 mg/day. Similar to patients in the RCT, cancer was the most common underlying advanced illness (57.0%), and the median daily morphine equivalent dose was 157.0 mg/day. During the OLE study, the mean (SD) and median (range) number of total injections per patient equaled 13.0 (11.3) and 10 (1–47), respectively.

### Efficacy

**FIG. 2.**
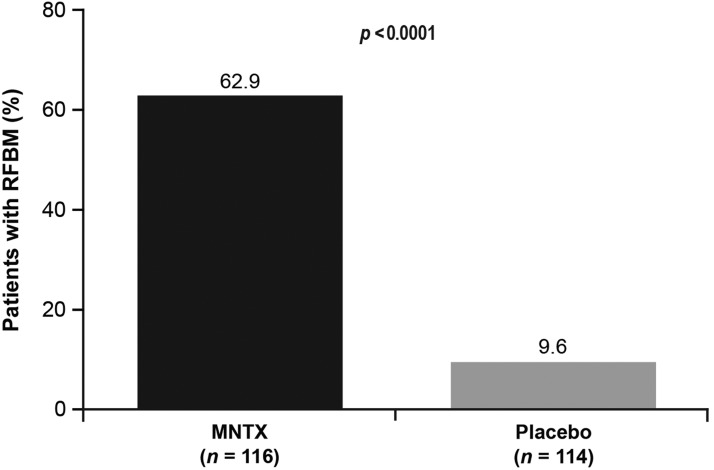
RFBM within four hours after ≥2 of the first 4 doses of MNTX or placebo in the first week of treatment in the RCT. MNTX, methylnaltrexone; RCT, randomized, placebo-controlled trial; RFBM, rescue-free bowel movement.

For the primary endpoint of the RCT, the percentage of patients who had an RFBM within four hours after ≥2 of the first 4 doses of study drug in the first week of treatment was 62.9% (95% confidence interval [CI], 53.5% to 71.7%) for the MNTX arm and 9.6% (95% CI, 4.9% to 16.6%) for the placebo arm (*p*<0.0001; see Fig. 2). Patient baseline weight (<62 kg versus ≥62 kg) did not affect the primary endpoint response of MNTX treatment compared with placebo (62.2% versus 7.3% for MNTX versus placebo in the <62 kg group, compared with 63.4% versus 11.0% for MNTX versus placebo in the ≥62 kg group; *p*<0.0001). Although there were few patients with a baseline body weight >114 kg, the primary endpoint response for this group (60.0% versus 0% for MNTX [*n*=5] versus placebo [*n*=5]; *p*=0.1667) was similar compared with that for patients with a baseline body weight ≤114 kg (63.1% versus 10.1% for MNTX [*n*=111] versus placebo [*n*=109]; *p*<0.0001).

**FIG. 3.**
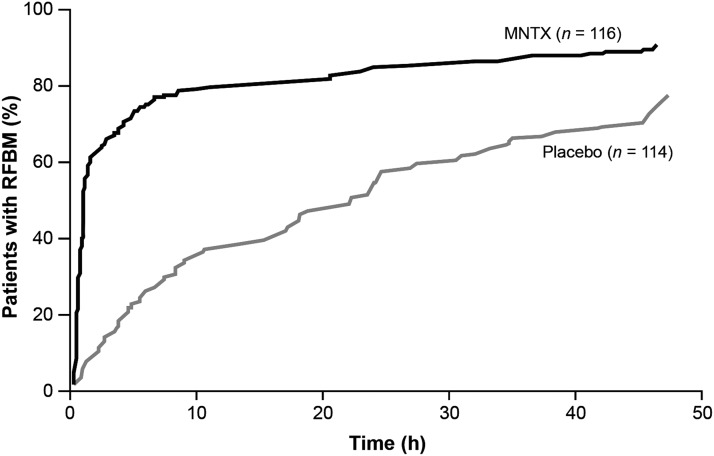
Kaplan-Meier curves for the time to bowel movement after first dose of the study drug in the MNTX and placebo groups in the RCT. MNTX, methylnaltrexone; RCT, randomized, placebo-controlled trial; RFBM, rescue-free bowel movement.

Significant differences favoring MNTX were observed for all secondary efficacy endpoints measured in the RCT (see Table 2). The time to RFBM after the first dose in the RCT was rapid in the MNTX group, with a median time of 0.8 hour versus 23.6 hours for the placebo group (*p*<0.0001; see Fig. 3). The median time to first RFBM after the first dose was similar in both weight groups (0.8 hour versus 24.5 hours for MNTX versus placebo in the <62 kg group compared with 0.8 hour versus 22.2 hours for MNTX versus placebo in the ≥62 kg group; *p*<0.0001). In addition, median times to first RFBM within 24 hours after each dose were significantly shorter in patients in the MNTX group compared with patients in the placebo group (*p*<0.005 after the first, third, fourth, fifth, sixth, and seventh dose). Efficacy during the 10-week OLE study (see Table 3) was generally consistent with results from the two-week RCT.

**Table 2.** Secondary Efficacy Endpoints in the RCT

*Endpoints*	*MNTX (*n*=116)*	*Placebo (*n*=114)*	p
Patients with first RFBM ≤4 hours after the first dose, *n*/*N* (%)	81/116 (69.8)	20/114 (17.5)	<0.0001
Patients with RFBM ≤4 hours after at least four of the maximum seven doses, *n*/*N* (%)	56/90 (62.2)	4/82 (4.9)	<0.0001
Mean number of BMs ≤24 hours after dosing (95% CI)			
Week 1	4.9 (4.3, 5.6)	3.0 (2.3, 3.7)	<0.0001
Week 2	3.2 (2.7, 3.7)	2.2 (1.7, 2.8)	0.0083
Mean number of RFBMs ≤24 hours after dosing (95% CI)			
Week 1	4.9 (4.2, 5.6)	2.7 (2.0, 3.4)	<0.0001
Week 2	3.2 (2.6, 3.7)	2.0 (1.5, 2.5)	0.0024
Patients using rescue laxatives in the RCT, *n*/*N* (%)	31/116 (27.2)	46/114 (39.6)	0.0020

BM, bowel movement; CI, confidence interval; MNTX, methylnaltrexone; RCT, randomized, placebo-controlled trial; RFBM, rescue-free bowel movement.

**Table 3.** Efficacy Endpoints in the OLE Study

	*MNTX PRN (*n*=147)^a^*
Number of BMs within 24 hours of dosing per patient per week, mean (SD)	
Range per week (minimum, maximum)	2.2 (1.6) to 3.1 (3.0)
Overall (10 weeks)	13.9 (15.9)
Number of days with BMs within 24 hours of dosing per patient per week, mean (SD), days	
Range per week (minimum, maximum)	1.7 (1.1) to 2.0 (1.6)
Overall (10 weeks)	9.6 (9.3)
Percentage of injections resulting in BM within 4 hours, mean (SD)	54.9 (33.4)

^a^ Two patients in the MNTX 12 mg group did not have diary data and were not included in the efficacy analyses.

BM, bowel movement; MNTX, methylnaltrexone; OLE, open-label extension; PRN, as needed; SD, standard deviation.

### Safety

In the RCT, the most common AEs in the MNTX group were abdominal pain and nausea (see Table 4). The most common AEs considered by the investigators to be possibly related to the study drug in the MNTX group were abdominal pain (28.4%), flatulence (6.0%), diarrhea (5.2%), and nausea (4.3%). The percentage of AEs that led to study discontinuation was 10.3% in the MNTX group and 6.1% in the placebo group. More serious AEs (SAEs) were reported in the placebo group (21.1% versus 12.1% in the MNTX group) with none of the SAEs considered related to the study drug by the investigator. Overall, there were 25 deaths in the RCT (11 and 14 in the MNTX and placebo groups, respectively); most deaths were attributed to underlying disease progression (9/11 and 13/14 deaths in the MNTX and placebo groups, respectively).

**Table 4.** Summary of Adverse Events During the RCT and the OLE Study

	*RCT*		*OLE study*
*Adverse event,* n *(%)*	*MNTX 8 mg or 12 mg QOD (*n*=116)*	*Placebo (*n*=114)*	*MNTX 8 mg or 12 mg PRN (*n*=149)*
SAE	14 (12.1)	24 (21.1)	59 (39.6)
Any AE	95 (81.9)	84 (73.7)	135 (90.6)
Drug-related AEs	49 (42.2)	21 (18.4)	38 (25.5)
Most common AEs^a^			
Abdominal pain	39 (33.6)	19 (16.7)	40 (26.8)
Nausea	13 (11.2)	18 (15.8)	21 (14.1)
Back pain	9 (7.8)	3 (2.6)	7 (4.7)
Diarrhea	9 (7.8)	15 (13.2)	24 (16.1)
Fall	9 (7.8)	4 (3.5)	21 (14.1)
Flatulence	8 (6.9)	5 (4.4)	7 (4.7)
Confusional state	7 (6.0)	9 (7.9)	23 (15.4)
Peripheral edema	7 (6.0)	4 (3.5)	26 (17.4)
Vomiting	5 (4.3)	10 (8.8)	10 (6.7)

^a^ Reported in >5% of patients in any group in the RCT and listed by most common AE during the RCT for the MNTX group.

AE, adverse event; MNTX, methylnaltrexone; OLE, open-label extension; PRN, as needed; QOD, every other day; RCT, randomized, placebo-controlled trial; SAE, serious adverse event.

In the OLE study, the most common AEs (>10% of patients) were abdominal pain, peripheral edema, diarrhea, fall, nausea, confusional state, asthenia, and dyspnea (see Table 4). AEs considered by the investigators to be at least possibly related to the study drug occurred in 25.5% of patients in the OLE study; the most common (reported in ≥5 patients) were abdominal pain (15.4%), diarrhea (7.4%), and flatulence (3.4%). AEs leading to study discontinuation were observed in 6.0% of patients, and SAEs occurred in 39.6% of patients, including 41 (27.5%) deaths. Most of the deaths (37 of 41 [90%]) were attributed to the progression of the underlying illness. None of the SAEs were considered study drug related by the investigator. Underlying disease progression was reported in 44 (29.5%) patients during the OLE study.

Patients in both RCT treatment groups had similar mean pain scores at baseline (4.0 in both groups) and showed minimal changes at day seven (3.5 and 3.8 for the MNTX and placebo groups, respectively). In the RCT, median opioid use during the study was comparable to baseline use for both the MNTX (180.0–164.6 mg morphine equivalents/day) and the placebo (160.8–127.1 mg morphine equivalents/day) groups. Median daily opioid use in the OLE study in morphine equivalent doses (121 mg/day) was comparable to baseline values at the start of the OLE study (157 mg/day).

## Discussion

The safety and efficacy of subcutaneous MNTX in the treatment of OIC in patients with advanced illness in hospice and palliative care settings was demonstrated in two pivotal phase 3 trials; however, these trials used weight-based dosing of MNTX.^17,18^ Because administering weight-based MNTX may be confusing and tedious for patients and caregivers to draw a variable dose of medication, using fixed-dose MNTX may be beneficial in this fragile patient population. The study presented here describes results from two phase 4 trials evaluating the safety and efficacy of fixed-dose subcutaneous MNTX to treat OIC in patients with advanced illness. Using baseline weight as a basis for dose selection, the fixed dose used in this study (8 mg and 12 mg MNTX) resulted in a range of exposures that were similar to the range of exposures observed during a study of 0.15 mg/kg in healthy volunteers of varying weight.^19^ In addition, the patients in these two trials were enrolled from a variety of health care settings, thereby providing safety and efficacy information on the use of fixed-dose MNTX in multiple health care situations.

Similar to the results from the phase 3 trials,^17,18^ fixed-dose MNTX administered QOD during the RCT demonstrated robust efficacy in treating OIC in patients with advanced illness. This patient population did not respond to a standard laxative regimen and continued to have OIC at baseline despite continued laxative use. Significantly superior efficacy was reported with MNTX compared with placebo for the primary and all secondary endpoints assessed in the RCT. The response to MNTX was rapid, with most patients achieving RFBMs within one hour of treatment. The benefit of shorter median times to RFBM with MNTX treatment compared with placebo persisted for all seven double-blind doses in the RCT. Patient baseline weight (<62 kg versus ≥62 kg) did not substantially affect the efficacy profile of MNTX versus placebo for the relief of OIC. Furthermore, the benefit of fixed doses of MNTX observed in the RCT was durable during the 10 weeks of the OLE study.

Fixed-dose MNTX demonstrated a favorable safety profile consistent with the approved labeling for this medication during this two-week RCT in patients with advanced illness.^20^ The tolerability profile of MNTX was also favorable in the 10-week OLE study. Similar to what was observed in the pivotal phase three trials,^17,18,21^ abdominal pain was the most common AE in patients treated with MNTX in both studies, which was mostly mild to moderate in intensity. Intentional initiative of propulsive peristalsis of the gut during normal bowel movement response induced by MNTX may be perceived as abdominal pain in some patients. SAEs were less common with MNTX than with placebo in this study. Consistent with previous studies in palliative care and noncancer pain, MNTX did not interfere with opioid-induced analgesia,^17,18,22^ reinforcing the concept of a peripherally restricted action of MNTX.

A limitation of the study is that the efficacy of fixed-dose MNTX in patients weighing <38 kg was not evaluated, because these patients were excluded from the trial. In addition, the RCT treatment phase was limited to a duration of two weeks, although data from the OLE study phase suggest that efficacy was maintained for a longer duration. In conclusion, subcutaneous fixed-dose MNTX administered QOD in the RCT and PRN in the OLE study demonstrated rapid and durable efficacy in the treatment of OIC in patients with advanced illness. The safety profile was favorable with no new safety issues identified with an MNTX dose of up to 12 mg/day for up to 12 weeks of treatment.
